# Supplementary material for: The mechanism of RNA capping by SARS-CoV-2
Source: Nature. 2022 Aug 9;609(7928):793–800. doi: 10.1038/s41586-022-05185-z (PMC9492545; doi:10.1038/s41586-022-05185-z)
Supplement: Supplementary file 1 — Supplementary Fig. 1: uncropped gels. Supplementary Tables 1–3: lists of RNAs used in the study; data collection and refinement statistics; and the oligonucleotides used in the SARS-CoV-2 infection experiments. [file 41586_2022_5185_MOESM1_ESM.pdf]

nature portfolio

https://doi.org/10.1038/s41586-022-05185-z

Supplementary information

The mechanism of RNA capping by SARS-CoV-2

In the format provided by the authors and unedited

Supplementary information

The Mechanism of RNA Capping by SARS-CoV-2

Gina J. Park[1†], Adam Osinski[1†], Genaro Hernandez[1], Jennifer L. Eitson[2], Abir Majumdar[1], Marco Tonelli[3], Katie Henzler-Wildman[3], Krzysztof Pawłowski[1,4], Zhe Chen[5], Yang Li[5], John W. Schoggins[2], Vincent S. Tagliabracci[1,6,7,8*]

*Correspondence to: Vincent S. Tagliabracci (vincent.tagliabracci@utsouthwestern.edu)

†These authors contributed equally to this work.

This file contains:

Supplementary Fig. 1, Uncropped gels

Supplementary Tables 1 to 3

Supplementary Fig. 1, Uncropped gels

Gel Figure 1a. ATP Coomassie

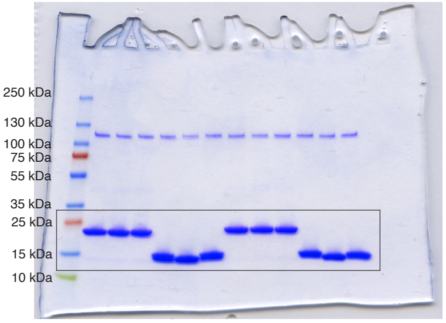

Gel Figure 1a. ATP Autorad

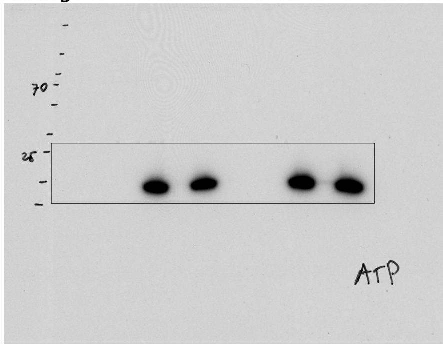

Gel Figure 1a. GTP Coomassie

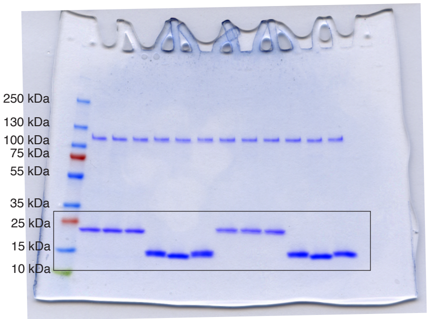

Gel Figure 1a. GTP Autorad

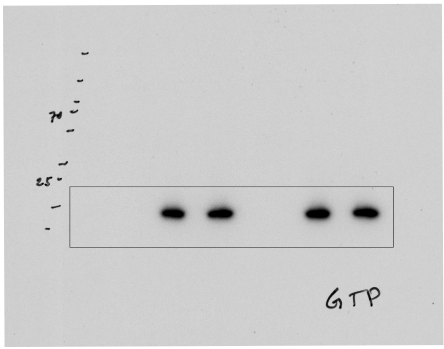

Gel Figure 1a. UTP Coomassie

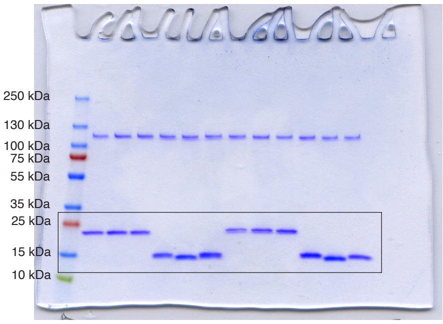

Gel Figure 1a. UTP Autorad

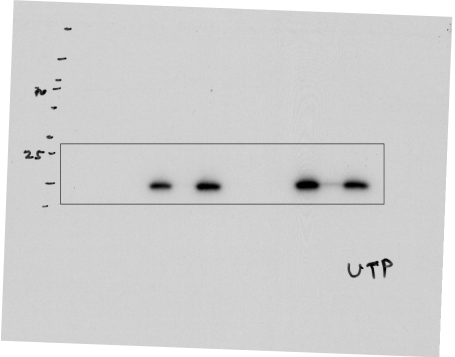

Gel Figure 1a. CTP Coomassie

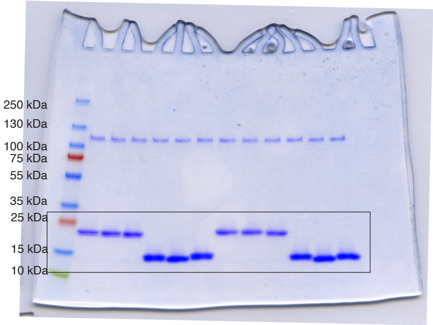

Gel Figure 1a. CTP Autorad

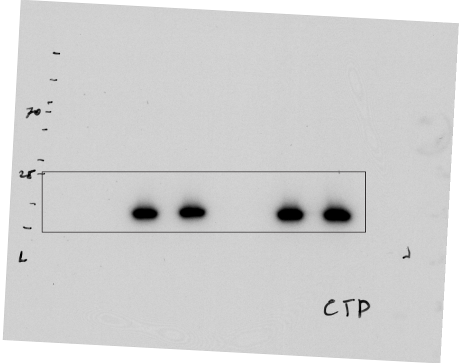

Gel Figure 1b. Coomassie

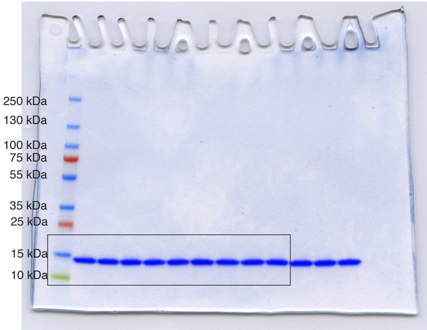

Gel Figure 1b. Autorad

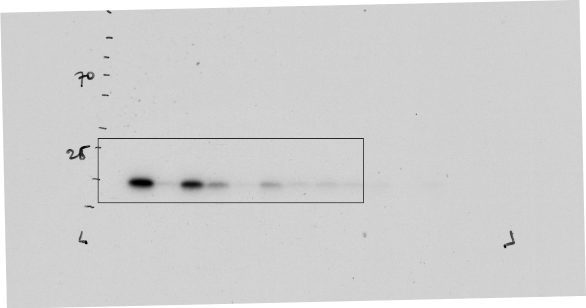

gel in Fig. 2b

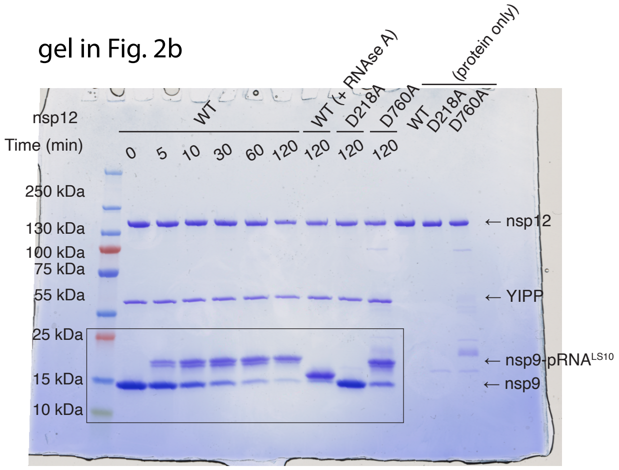

gel in fig. 3b

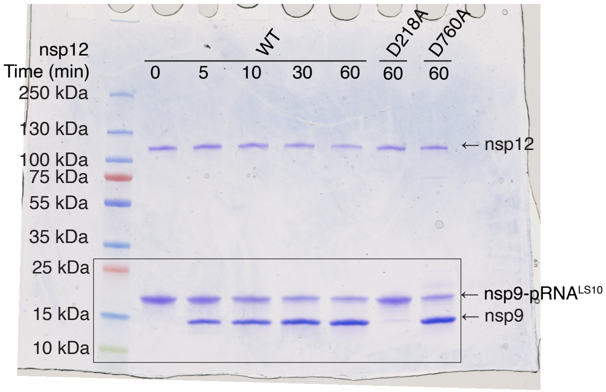

gel in Fig. 2c

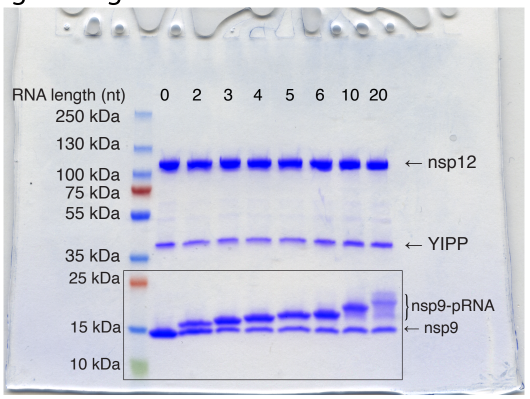

gel inFig. 3c

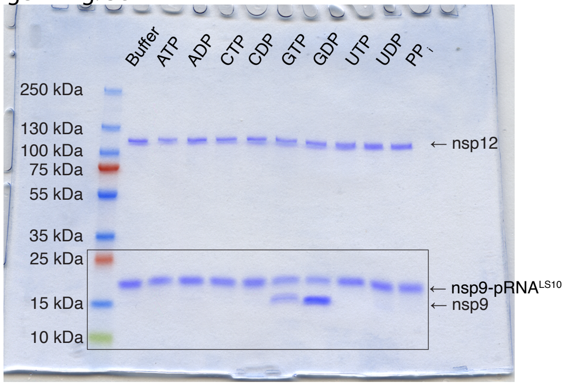

gel in Fig. 2d

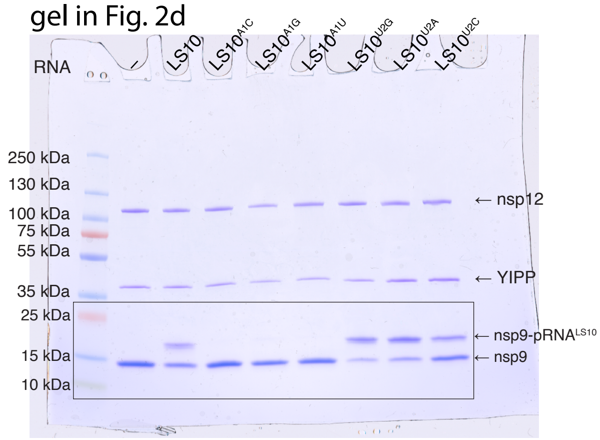

gel in fig. 3a

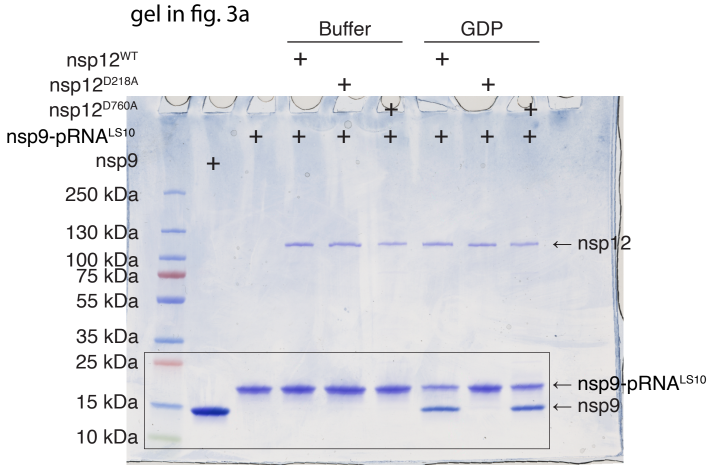

gel in Fig. 3d

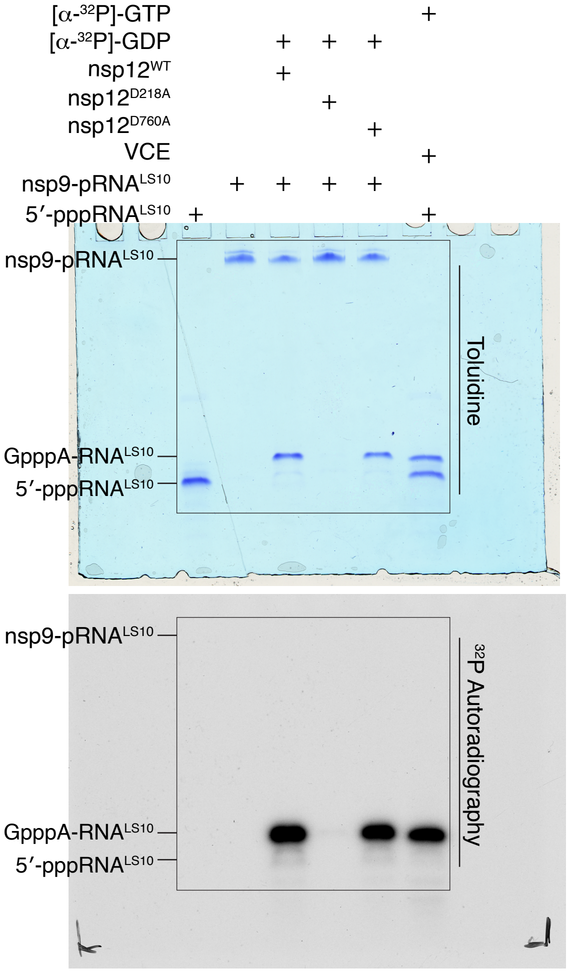

4a

Thin Layer Chromatography

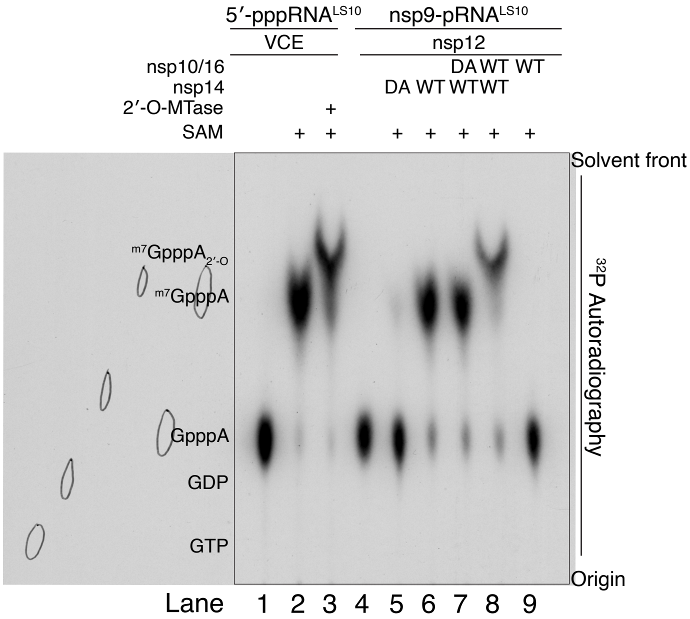

Gel Extended Figure 3a. GTP Coomassie

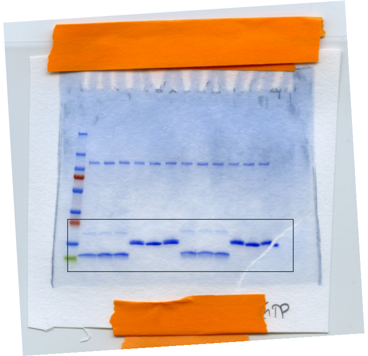

Gel Extended Figure 3a. GTP Autorad

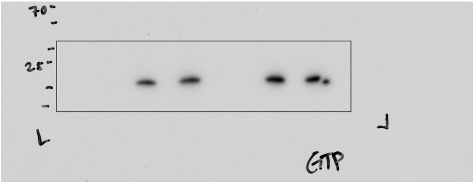

Gel Extended Figure 3a. UTP Coomassie

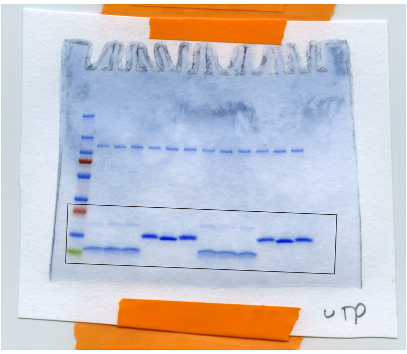

Gel Extended Figure 3a. UTP Autorad

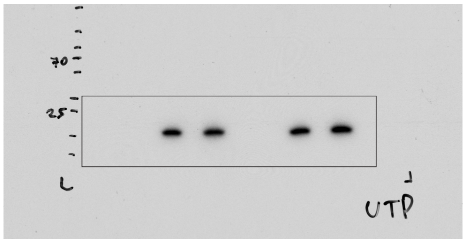

Gel Extended Data Fig. 4b

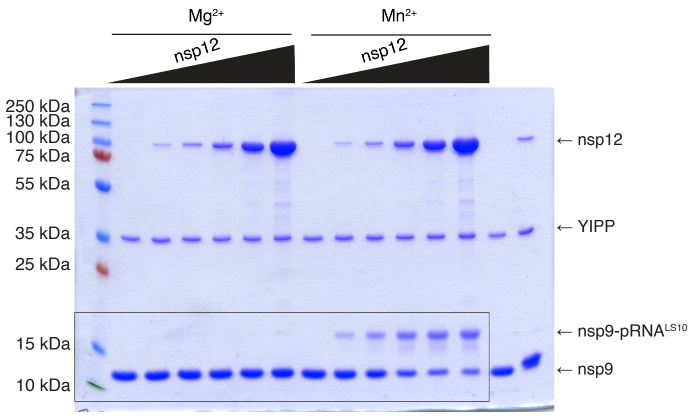

Gel Extended Data Fig. 4c

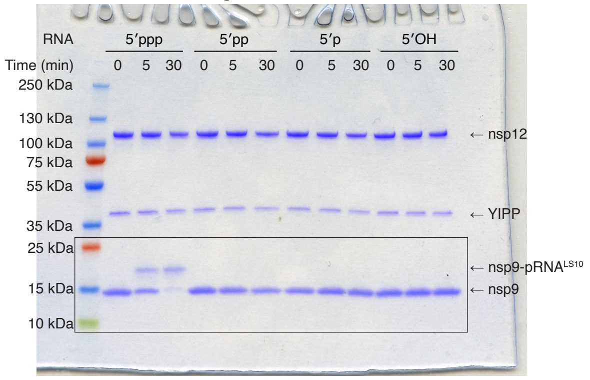

gel Extended Data fig. 4d

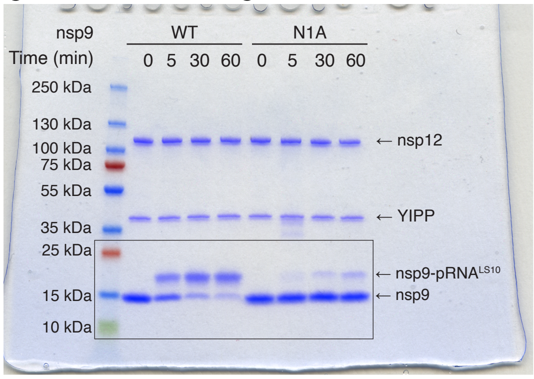

Gel Extended Data Fig. 5c

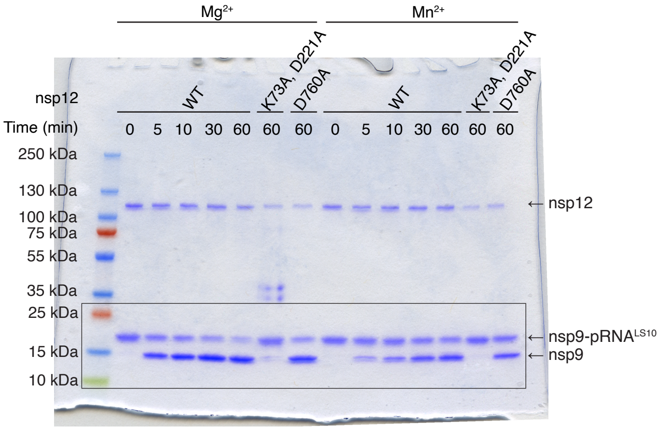

gel Extended Data fig. 5d

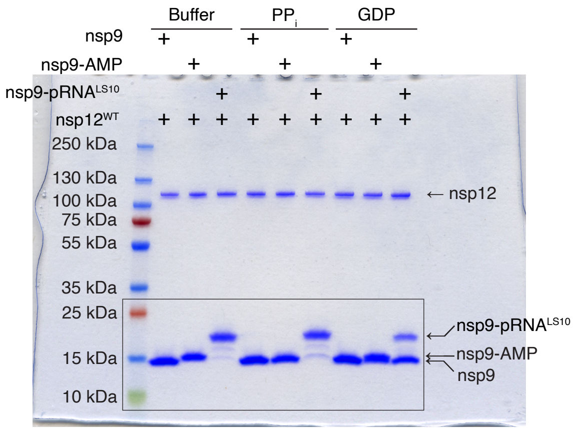

Extended Data Fig. 6a

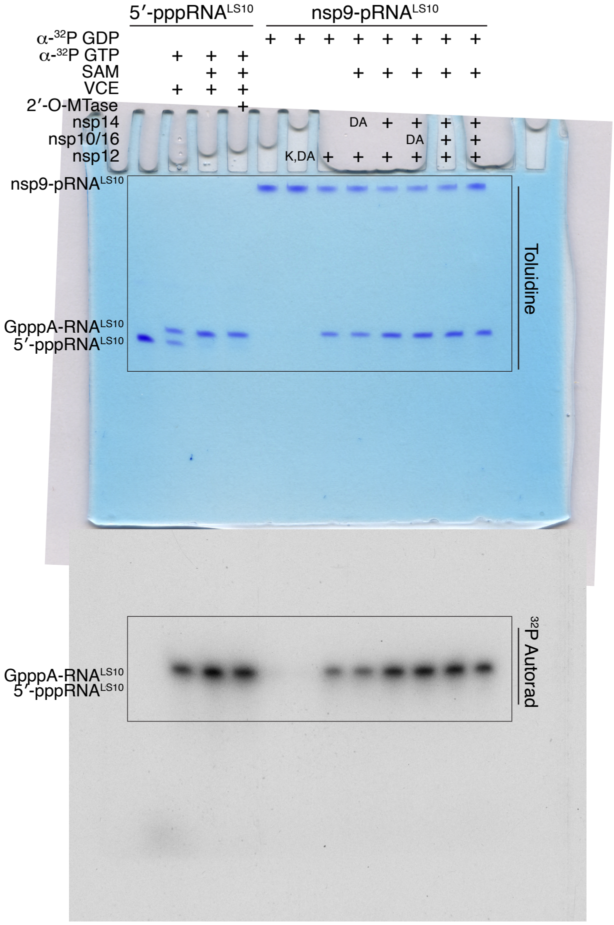

Extended Data 5g

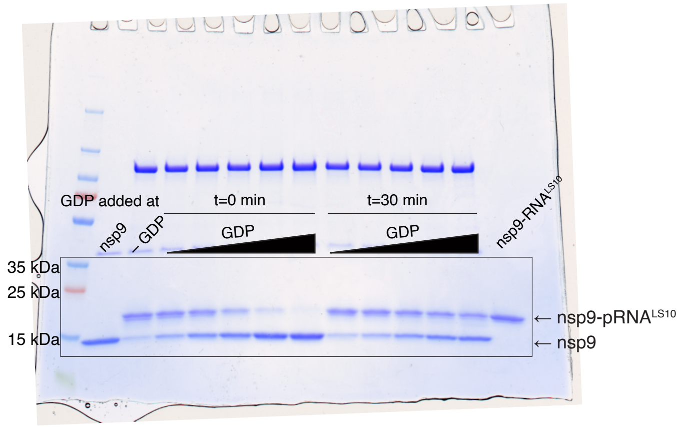

Extended Data 5h

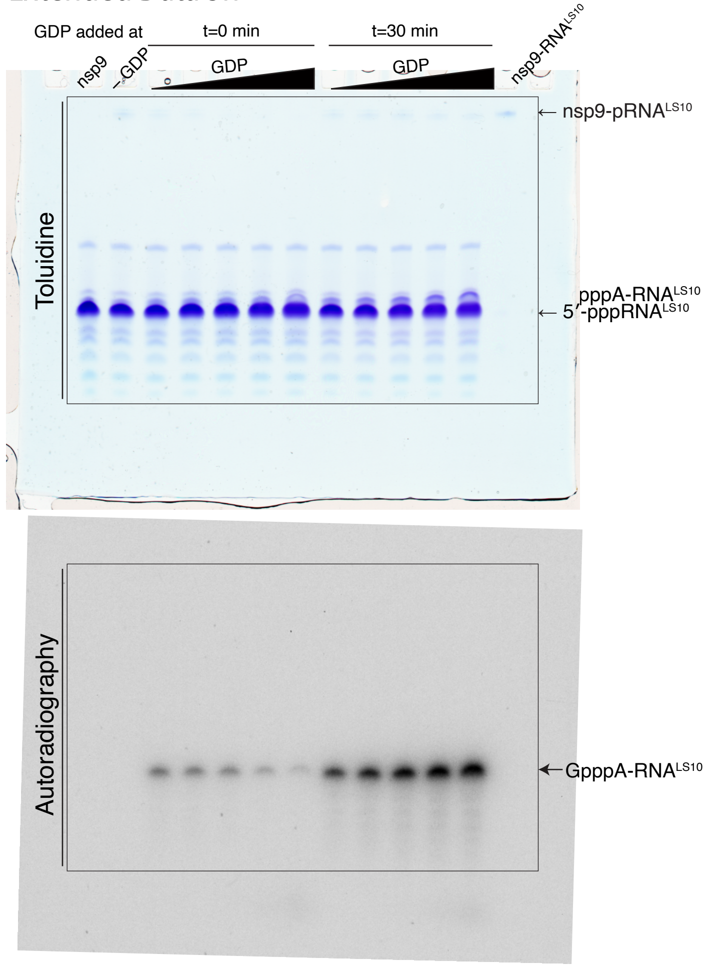

Extended Data Fig. 6b

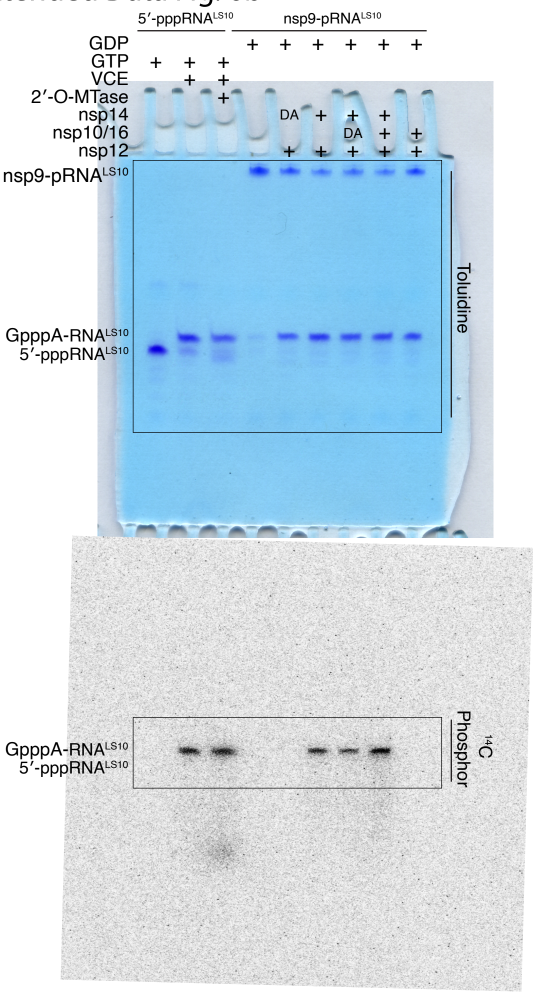

Gel Extended Data Fig. 8b

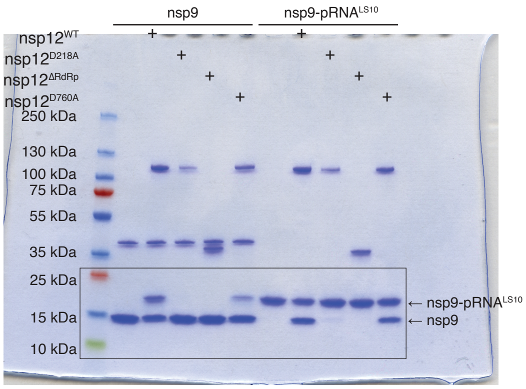

Gel Extended Figure 10d. Toluidine

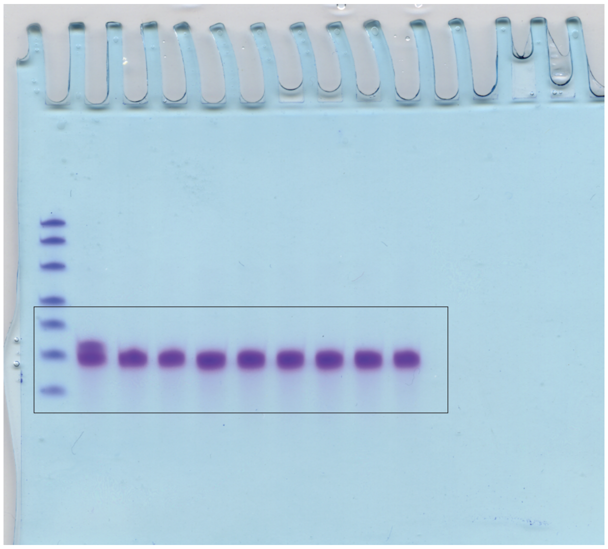

Extended Data 9d

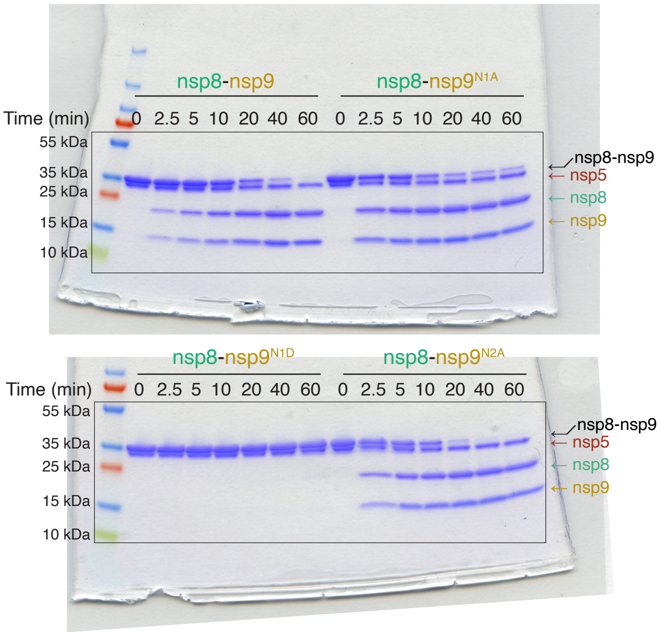

Gel Extended Figure 10d. Autorad

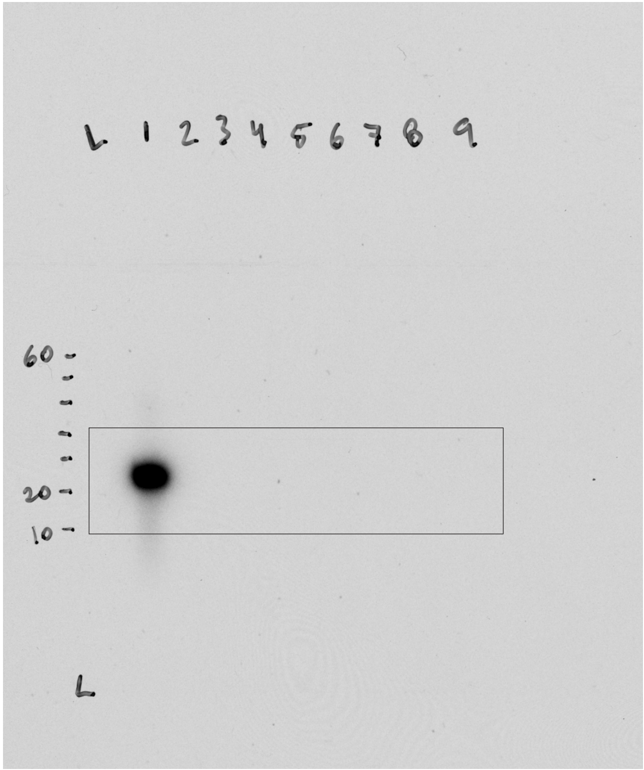

Supplementary Table 1. RNAs used in this study

**RNA**	**Sequence**
5′-pppRNA^{A19C} [26]	[ppp]ACCCCCCCCCCCCCCCCCCC
5′-pppRNA^{LS10}	[ppp]AUUAAAGGUU
5′-pppRNA^{LS2}	[ppp]AU
5′-pppRNA^{LS3}	[ppp]AUU
5′-pppRNA^{LS4}	[ppp]AUUA
5′-pppRNA^{LS5}	[ppp]AUUAA
5′-pppRNA^{LS6}	[ppp]AUUAAG
5′-pppRNA^{LS20}	[ppp]AUUAAAGGUUUAUACCUUCC
5′-pppRNA^{LS10_A1C}	[ppp]CUUAAAGGUU
5′-pppRNA^{LS10_A1G}	[ppp]GUUAAAGGUU
5′-pppRNA^{LS10_A1U}	[ppp]UUUAAAGGUU
5′-pppRNA^{LS10_U2G}	[ppp]AGUAAAGGUU
5′-pppRNA^{LS10_U2A}	[ppp]AAUAAAGGUU
5′-pppRNA^{LS10_U2C}	[ppp]ACUAAAGGUU

Supplementary Table 2. Data collection and refinement statistics

Magnification	81,000
Voltage (kV)	300
Electron exposure (e^{-}Å^{-2})	54
Defocus range (μm)	-1.0 to -2.5
Pixel size (Å)	1.09
Symmetry imposed	C1
Initial particle images (no.)	4,196,806
Final particle images (no.)	39,985
Map resolution (Å)	3.2
FSC threshold	0.143
**Refinement**	
Initial model used (PDB code)	-
Model resolution (Å)	3.5
FSC threshold	0.5
Map sharpening *B* factor (Å^{-2})	-37
Nonhydrogen atoms	9,215
Protein residues	1150
Ligands	4
B factors (Å^{-2})	
Protein	67.4
Ligands	75.9
R.m.s. deviations	
Bond lengths (Å)	0.002
Bond angles (˚)	0.453
MolProbity score	1.50
Clashscore	3.12
Poor rotamers (%)	0
Favored (%)	94.1
Allowed (%)	5.9
Disallowed (%)	0

Supplementary Table 3. Oligonucleotides used in the SARS-CoV-2 infection experiments

Oligo	Sequence	Use
PacI Forward	GGTTGAAGCAGTTAATTAAAGTTACACTTGTG	Fragment 1 and 3
1NtoA Reverse	GCAACAGGACTAAGCTCATTAGCCTGTAATTTGACAGC	Fragment 1
1NtoD Reverse	GCAACAGGACTAAGCTCATTGTCCTGTAATTTGACAGC	Fragment 1
2NtoA Reverse	GCAACAGGACTAAGCTCAGCATTCTGTAATTTGACAGC	Fragment 1
73KtoA Reverse	GAGAAAGTGTGTCTCGCAACTACAAAGTAAG	Fragment 1
MluI Reverse	CCTAAGTTGGCGTATACGCGTAATATATCTGGG	Fragment 2 and 3
1NtoA Forward	GCTGTCAAATTACAGGCTAATGAGCTTAGTCCTGTTGC	Fragment 2
1NtoD Forward	GCTGTCAAATTACAGGACAATGAGCTTAGTCCTGTTGC	Fragment 2
2NtoA Forward	GCTGTCAAATTACAGAATGCTGAGCTTAGTCCTGTTGC	Fragment 2
73KtoA Forward	CTTACTTTGTAGTTGCGAGACACACTTTCTC	Fragment 2
