# Supplementary material for: The mechanism of RNA capping by SARS-CoV-2
Source: Nature. 2022 Aug 9;609(7928):793–800. doi: 10.1038/s41586-022-05185-z (PMC9492545; doi:10.1038/s41586-022-05185-z)
Supplement: Supplementary file 2 — Reporting Summary [file 41586_2022_5185_MOESM2_ESM.pdf]

nature portfolio

Corresponding author(s): Vincent S. Tagliabracci

Last updated by author(s): Jul 25, 2022

Reporting Summary

Nature Portfolio wishes to improve the reproducibility of the work that we publish. This form provides structure for consistency and transparency in reporting. For further information on Nature Portfolio policies, see our Editorial Policies and the Editorial Policy Checklist.

Statistics

For all statistical analyses, confirm that the following items are present in the figure legend, table legend, main text, or Methods section.

n/a	Confirmed	
☐	☒	The exact sample size (*n*) for each experimental group/condition, given as a discrete number and unit of measurement
☐	☒	A statement on whether measurements were taken from distinct samples or whether the same sample was measured repeatedly
☒	☐	The statistical test(s) used AND whether they are one- or two-sided<br>*Only common tests should be described solely by name; describe more complex techniques in the Methods section.*
☒	☐	A description of all covariates tested
☒	☐	A description of any assumptions or corrections, such as tests of normality and adjustment for multiple comparisons
☐	☒	A full description of the statistical parameters including central tendency (e.g. means) or other basic estimates (e.g. regression coefficient) AND variation (e.g. standard deviation) or associated estimates of uncertainty (e.g. confidence intervals)
☒	☐	For null hypothesis testing, the test statistic (e.g. *F*, *t*, *r*) with confidence intervals, effect sizes, degrees of freedom and *P* value noted<br>*Give P values as exact values whenever suitable.*
☒	☐	For Bayesian analysis, information on the choice of priors and Markov chain Monte Carlo settings
☒	☐	For hierarchical and complex designs, identification of the appropriate level for tests and full reporting of outcomes
☒	☐	Estimates of effect sizes (e.g. Cohen's *d*, Pearson's *r*), indicating how they were calculated

Our web collection on statistics for biologists contains articles on many of the points above.

Software and code

Policy information about availability of computer code

Data collection	1) Cryo-EM: SerialEM 4.0.4 (ref 46).
Data analysis	1) NMR: All 2D spectra were processed using NMRPipe 11.0 (ref. 42) and analysed with NMRFAM-SPARKY 1.3 (ref 43).<br>2) Intact mass spectrometry: The acquired mass spectra for the proteins of interest were deconvoluted using BioPharmaView v. 3.0.1 software (Sciex) in order to obtain the molecular weights.<br>3) HPLC/MS: Peak areas were determined and data were further analysed using the Sciex Analyst 1.7.2 software package.<br>4) GraphPad Software 9.4.0 , San Diego, California USA, www.graphpad.com<br>5) Cryo-EM: Relion 3.1 (ref. 47); MotionCor2 1.5 (ref. 48); Gctf 1.06 (ref. 49); crYOLO 1.7 (ref. 50); cryoSPARC 3.2 (ref. 51); Coot 0.9.6 (ref. 52); Phenix 1.20.1-4487 (ref. 53); MolProbity 4.5.1 (ref. 54).<br>6) Bioinformatics: FATCAT 2.0 (ref. 55); ESPript 3 (ref. 56)<br>7) Fiji 2.1.0/1.53h (ref. 44)

For manuscripts utilizing custom algorithms or software that are central to the research but not yet described in published literature, software must be made available to editors and reviewers. We strongly encourage code deposition in a community repository (e.g. GitHub). See the Nature Portfolio guidelines for submitting code & software for further information.

Data

Policy information about availability of data

All manuscripts must include a data availability statement. This statement should provide the following information, where applicable:

- Accession codes, unique identifiers, or web links for publicly available datasets
- A description of any restrictions on data availability
- For clinical datasets or third party data, please ensure that the statement adheres to our policy

Cryo-EM model has been deposited in the Protein Data Bank under accession number 7THM. Cryo-EM density maps have been deposited in the Electron Microscopy Data Bank with accession codes EMD-25898. All other data generated during and/or analysed during the current study are available from the corresponding author on reasonable request.
Additionally, publicly available datasets were used:
Pseudomonas syringae SelO protein crystal structure PDBID: 6EAC
Mouse PKA C-alpha crystal structure PDBID: 1ATP
Extended SARS-CoV-2 RTC complex structure with non-native nsp9 PDBID: 7CYQ, EMDB: EMD-30504

Field-specific reporting

Please select the one below that is the best fit for your research. If you are not sure, read the appropriate sections before making your selection.

☒ Life sciences ☐ Behavioural & social sciences ☐ Ecological, evolutionary & environmental sciences

For a reference copy of the document with all sections, see nature.com/documents/nr-reporting-summary-flat.pdf

Life sciences study design

All studies must disclose on these points even when the disclosure is negative.

Sample size	No sample size was predetermined. Sample sizes were chosen according to the standard generally accepted in the field.
Data exclusions	No data were excluded from the analyses.
Replication	Each experiment presented in the paper was repeated at least twice, with similar results. Reported results were consistently replicated across all experiments.
Randomization	No randomization was used in performing in vitro biochemical experiments. Covariate control is irrelevant for this study. No human or animal subjects were used in the study.
Blinding	Blinding was not used in this study. Due to the experimental setup of the in-vitro experiments, blinding was not possible, or would not provide benefit.

Reporting for specific materials, systems and methods

We require information from authors about some types of materials, experimental systems and methods used in many studies. Here, indicate whether each material, system or method listed is relevant to your study. If you are not sure if a list item applies to your research, read the appropriate section before selecting a response.

Materials & experimental systems

n/a	Involved in the study	
☒	☐	Antibodies
☐	☒	Eukaryotic cell lines
☒	☐	Palaeontology and archaeology
☒	☐	Animals and other organisms
☒	☐	Human research participants
☒	☐	Clinical data
☒	☐	Dual use research of concern

Methods

n/a	Involved in the study	
☒	☐	ChIP-seq
☒	☐	Flow cytometry
☒	☐	MRI-based neuroimaging

Eukaryotic cell lines

Policy information about cell lines

Cell line source(s)	BHK-21J cells were a generous gift from C. Rice, not commercially sourced. VeroE6-C1008 cells were obtained from ATCC.

Authentication	These are not human cells and thus we do not do STR profiling. The ATCC authenticates VeroE6-C1008. BHK-21J were not authenticated.
Mycoplasma contamination	All cell lines are routinely tested for mycoplasma using a sensitive PCR-based assay. The cells in this study tested negative for mycoplasma.
Commonly misidentified lines (See ICLAC register)	No commonly misidentified lines were used.
